# Risk of perioperative stroke and cerebral autoregulation monitoring: a systematic review

**DOI:** 10.1055/s-0042-1758648

**Published:** 2022-12-29

**Authors:** Daniel Paes de Almeida dos Santos, Parthasarathy Deenadayalan Thirumala, Gautama Reddy, Daniel Ferreira de Barros, Vinicius Naves Rezende Faria, Varun Shandal, Pedro Kurtz

**Affiliations:** 1 Hospital Copa Star, Centro de Terapia Intensiva, Rio de Janeiro RJ, Brazil.; 2 Sinapse Clínica, Rio de Janeiro RJ, Brazil.; 3 Intraneuro, Rio de Janeiro RJ, Brazil.; 4 University of Pittsburgh Medical Center, Pittsburgh, Pennsylvania, United States.; 5 Universidade Federal de Uberlândia, Faculdade de Engenharia Elétrica, Uberlândia MG, Brazil.; 6 Instituto D'Or de Pesquisa e Ensino, Departamento de Cuidado Intensivo, São Paulo SP, Brazil.

**Keywords:** Ultrasonography, Doppler, Transcranial, Heart Bypass, Right, Spectroscopy, Near-Infrared, Cerebrovascular Circulation, Ultrassonografia Doppler Transcraniana, Derivação Cardíaca Direita, Espectroscopia de Luz Próxima ao Infravermelho, Circulação Cerebrovascular

## Abstract

**Background** Perioperative stroke, delirium, and cognitive impairment could be related to management and to variations in blood pressure control, cerebral hypoperfusion and raised blood volume. Cerebral autoregulation (CAR) is a mechanism to maintain cerebral perfusion through the control of the vascular tone and hemodynamic reactions in the circulation.

**Objective** The present systematic review addresses the relationship between impaired CAR and perioperative stroke by evaluating the rate of neurological complications after surgery in studies in which perioperative CAR was tested or monitored.

**Methods** We included randomized clinical trials and prospective observational studies. All studies had adjusted the relative risk, hazard ratio or 95% confidence interval (95%CI) values. These estimation effects were tested using random-effects models. Heterogeneity among the selected studies was assessed using the Higgins and Thompson I ^2^ statistics.

**Results** The Web of Science, PubMed and EMBASE electronic databases were searched to retrieve articles. A total of 4,476 studies published between 1983 and 2019 were analyzed, but only 5 qualified for the data extraction and were included in the final analysis. The combined study cohort comprised 941 patients who underwent CAR monitoring during surgical procedures. All studies provided information about perioperative stroke, which equated to 16% (158 of 941) of the overall patient population.

**Conclusion** The present meta-analysis showed evidence of the impact of CAR impairment in the risk of perioperative stroke. On the pooled analysis, blood fluctuations or other brain insults large enough to compromise CAR were associated with the outcome of stroke (odds ratio [OR]: 2.26; 95%CI: 1.54–2.98; *p* < 0.0001).

## Resumo

**Antecedentes** Acidente vascular cerebral (AVC) perioperatório, delírio e comprometimento cognitivo podem estar relacionados ao manejo e à variações no controle da pressão arterial, à hipoperfusão cerebral, e ao aumento do volume sanguíneo. A autorregulação cerebral (ARC) é um mecanismo para manter a perfusão cerebral por meio do controle do tônus vascular e das reações hemodinâmicas na circulação.

**Objetivo** Este trabalho aborda sistematicamente a determinação da relação entre a falha da ARC e o AVC perioperatório, com uma avaliação da taxa de complicações neurológicas pós-cirúrgicas em estudos em que a ARC perioperatória foi monitorada.

**Métodos** Esta revisão sistemática incluiu ensaios clínicos randomizados e estudos observacionais prospectivos. Todos os estudos ajustaram o risco relativo, a razão de risco ou os valores de intervalo de confiança de 95% (IC95%). Esses efeitos de estimativas foram testados usando modelos de efeitos aleatórios. A heterogeneidade entre os diferentes estudos foi avaliada por meio das estatísticas de Higgins e Thompson I ^2^ .

**Resultados** As bases de dados eletrônicas Web of Science, PubMed e Embase foram pesquisadas para selecionar os artigos. Um total de 4.476 estudos publicados entre 1983 e 2019 foram analisados, mas apenas 5 se qualificavam para a extração de dados e foram incluídos na análise final. A coorte combinada do estudo foi composta por 941 pacientes. Todos os estudos forneceram informações sobre AVC perioperatório, o que equivaleu a 16% (158 de 941) da população geral de pacientes.

**Conclusão** Esta meta-análise mostrou evidências do impacto do prejuízo da ARC no risco de AVC perioperatório. Na análise conjunta, as flutuações sanguíneas ou outros insultos cerebrais grandes o suficiente para comprometer a ARC foram associados ao resultado do AVC (razão de probabilidades [RP]: 2,26; IC95%: 1,54–2,98; *p* < 0,0001).

## INTRODUCTION

Postoperative neurologic complications (PNCs), including stroke, delirium, and cognitive impairment, account for a significant increase in mortality and morbidity. 1 The rate of PNCs is variable and related to the type of surgical procedure, the preexistence of comorbidities, the intraoperative management, and the reporting guidelines. 1 2 3 These complications can be related to the perioperative control of blood pressure (BP), cerebral hypoperfusion, and raised blood volume. These variations are balanced by changes in vascular tone and hemodynamic reactions in the intracranial circulation to maintain adequate cerebral perfusion. 4 5 Due to a wide range of arterial blood pressures, cerebral blood flow (CBF) is relatively constantly controlled by a mechanism called cerebral autoregulation (CAR), 6 7 which, in turn, is dependent on metabolic, myogenic and neuronal mechanisms, including the reactions based on autonomic response. Therefore, is important being able to adequately maintain CBF considering cerebral metabolic demands. 7 8 because these mechanisms protect the brain fromoligemia or hyperemia. 9 In general, CAR refers to the ability to maintain adequate brain perfusion despite BP fluctuations, influencing the intracranial vascular tone and hemodynamic responses. 2 3 In healthy non-hypertensive individuals, brain perfusion can be maintained with regular mean BP variation in the range from 50 mmHg to 150 mmHg due to CAR. 10 Impaired CAR can cause cerebral hypo- or hyperfusion intraoperatively, and this has been considered one of the potential causes of major PNCs, mainly stroke. 4 The other reported causes of PNCs are low oxygenation, systemic inflammation, proinflammatory cytokines, 5 and anesthetic agents. 11

Recent studies have addressed the potential reasons for perioperative cerebral injury, such as the use of cardiopulmonary bypass (CPB) during cardiac surgeries, 8 but this has not been confirmed by other studies. 12 Disturbances in CAR mechanisms have already been linked with others types of brain injuries and outcomes. 13 14 Transcranial Doppler (TCD) and near-infrared spectroscopy (NIRS) are the two most used tools to monitor CAR during surgical procedures. 1 11 15 The temporal resolution and the sensitivity to detect small changes in CBF or cerebral oxygenation are fundamental qualities to indicate their use in monitoring. 14 16 The primary aim of the present study was to systematically review the current scientific literature and perform a meta-analysis to determine the relationship between impaired CAR and perioperative stroke. 17 We expect the results of the study to underscore that the need for CAR should be evaluated before a surgical procedure as a means of classifying the risk of perioperative stroke and delirium and/or cognitive function.

## METHODS

### Search criteria

We included different types of study, from randomized clinical trials to small prospective observational studies., were the source of those studies These studies were searched by the authors in major literature databases, including PubMed, Web of Science, and Cochrane, using the following MeSH terms: *surgery* ; *procedures* ; *intervention* ; *cerebral autoregulation* ; *cerebral hemodynamics* ; *cerebral reserve* ; *cerebral blood flow regulation* ; and *cerebrovascular circulation* .

The studies included different methods of CAR estimation and monitoring, although the most used were the mean flow index (Mx), which is based on the monitoring of the velocity of the middle cerebral artery (MCA) through TCD, and the cerebral oxygenation index (COx), based on cerebral oxygenation monitoring through NIRS. Publications on validation methods, as well as meta-analysis and systematic reviews, were not included. Studies which included patients with acute brain injury or in which the data regarding the outcome events could not be extracted were excluded from the analysis.

Duplicate articles in different search platforms, methodological flaws, failure to present clear methods to evaluate the CAR, lack of data necessary for the monitoring analysis or measurement of results, and lack of data regarding stroke as a clinical outcome were the complementary criteria for the exclusion of works. The objective of the present meta-analysis was to study the relationship between perioperative impaired CAR and stroke in elective surgeries.

The present systematic review and meta-analysis was conducted and reported according to the recommendations of the Meta-analysis of Observational Studies in Epidemiology (MOOSE) reporting guidelines. 18

### Study selection

The authors independently searched and identified the articles selected for review, which included CAR monitoring during surgeries and postoperative neurologic outcomes, and had reported the adjusted risk estimation for the 95% confidence interval (95%CI). Different studies in the same dataset encouraged the authors to choose the article which included the largest number of patients.

The selected studies involved CAR monitoring during the perioperative period. This usually lasts from the time the patient goes into the hospital or doctor's office for surgery until the time the patient goes home.

### Data extraction and quality assessment

Data were independently extracted by two authors (DPAS and GR) and submitted to a standardized form. Disagreements between the authors on matters such as selection of studies, outcome analysis, and potential impact of heterogeneity was decided through consensus. The inclusion criteria were: articles published in English on randomized controlled trials, or on prospective or retrospective cohort studies conducted with adult subjects and containing an abstract and the use of known monitoring strategies. The full text of the potentially relevant papers was then screened for inclusion in the final analysis. The data extracted from these studies included sample size, baseline characteristics, main interventions, and combined outcome events. Odds Ratios (ORs), 95% Cis, and other information used in the multivariate analysis were extracted. The quality of the studies was assessed by the Newcastle-Ottawa Scale. 18

### Statistical analysis

The studies included presented heterogeneity as an important factor to be considered. Therefore, the statistical analysis models were chosen based on these considerations. Initially, the studies were only considered for evaluation if they contained the adjusted relative risk, hazard ratio, or 95%CI values, which represent the best values corrected based on baseline characteristics. These estimation effects were tested using random-effects models. The fixed-effects models start with the assumption that the true effect is the same in all studies, but this assumption is implausible in our systematic review. So, random-effects models were chosen by the assumption of the number of studies and data modelling characteristics. The heterogeneity among the studies was assessed using Higgins and Thompson I ^2^ statistics. The proportion of total variation observed among the studies that is attributable to differences between them rather than to sampling errors (chance), with values (> 50%) corresponding to moderate levels of heterogeneity. All analyses were performed using the R statistical software (R Foundation for Statistical Computing, Vienna, Austria), version 4.1.1.

## RESULTS

### Overall analysis

**Figure 1:**
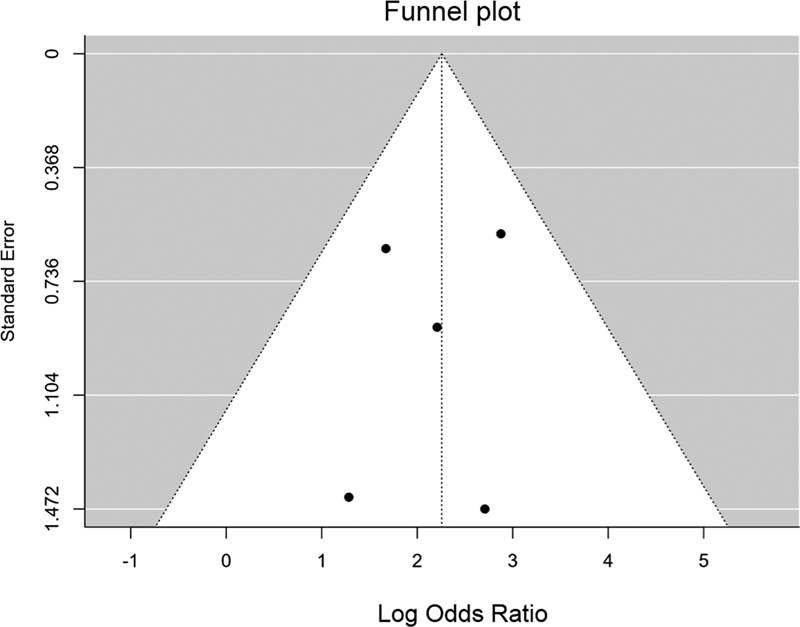
Flowchart of the selection of studies for the systematic review and summary report of the noted results.

The search on the Web of Science, PubMed, and EMBASE electronic databases yielded a total of 4,476 published studies on patients submitted to intraoperative CAR monitoring. The reviewed studies are shown on a flowchart in Figure 1 , together with the screening process; they were published between October 1983 and September 2019 and met the inclusion criteria. After removing 2,335 duplicates, 2,141 studies were screened based on their abstracts, and 2,059 were then excluded due to methodological flaws and for lacking to provide the necessary data for analysis on monitoring or outcome measurements. Therefore, 82 studies had their full texts reviewed, and only 5 qualified for the data extraction and were included in the final analysis. 19 20 21 22 23 24 They were mainly prospective studies (only one study was a randomized controlled clinical trial), and comprised 941 patients submitted to intraoperative CAR monitoring. The characteristics of the studies are summarized in Table 1 .

**Table 1:** Summary of the baseline characteristics of the patients enrolled in the studies analyzed

		Combined sample (n = 941)
Mean age (years)		65 (±1)
Sex: n (%)	Male	718 (76%)
	Female	221 (24%)
Medical history: n (%)	Hypertension	722 (76.7%)
	Diabetes	341 (36.2%)
	COPD	95 (10.1%)
Type of surgery: n (%)	CABG	560 (59.5%)
	CABG and valve	116 (12.3%)
	Valve	210 (22.3%)
	Thoracic aortic	3 (0.3%)
	CEA/CAS	35 (3.7%)
	Others	17 (1.8%)
Instrumentation: n (%)	TCD	396 (42.1%)
	NIRS	779 (82.8%)
	invasive BP	906 (96.3%)
	non-invasive BP	35 (3.16%)
	COx	671 (71.3%)
	TFA	35 (3.7%)
	Mx	361 (38.3%)
	MAP	906 (96.2%)
	CBF	234 (24.8%)
	ARI	108 (11.4%)
Outcomes: n (%)	Stroke	154 (16.37%)
	Mortality	16 (1.7%)

Abbreviations: ARI, autoregulation index; BP, blood pressure; CABG, coronary artery bypass graft; CAS, carotid balloon angioplasty with stenting; CEA, carotid endarterectomy; COPD, chronic obstructive pulmonary disease; COx, cerebral oxygenation index; MAP, mean arterial pressure; NIRS, near-infrared spectroscopy; TCD, transcranial Doppler; TFA, transfer function analysis, Mx, mean flow index.

### Diagnostic accuracy of the primary aim: intraoperative impaired CAR

Of the 941 patients in question, 76% (718) were male, with a mean age of 65 (standard deviation [SD]: ± 1.0) years. All studies provided information about perioperative stroke, which equated to 16% (158) of the overall patient population. Of this subset, only 1 of the studies 22 provided information about mortality, with a rate of 1.7% (16). In patients who underwent CAR monitoring for elective surgeries, most procedures were cardiac (886; 94%) and the most associated comorbidity was hypertension (722; 77%), followed by diabetes (341; 36%) and chronic obstructive pulmonary disease (COPD: 95; 10%). Regarding the country of origin, 2 studies were conducted in the United States, 21 22 1, in Australia, 24 and 2, in Europe. 20 24 In all studies, the patients were submitted to elective surgical procedures, mainly cardiac or carotid surgery with perioperative CAR monitoring. The method used to determine the CAR and the time in which it was performed in each paper are described in Table 2 .

**Table 2:** Summary of cerebral autoregulation monitoring in each selected study

Papers	Modalities	Summary	Period
Joshi et al. 20 (2010)	Monitoring through transcranial Doppler (TCD) of the blood flow velocity of the right and left middle cerebral arteries (MCAs)	Patients undergoing cardiopulmonary bypass (CPB) during cardiac surgery were submitted to TCD monitoring of the right and left MCAs. A continuous, moving Pearson correlation coefficient was calculated regarding the mean arterial blood pressure (MAP) and TCD blood flow velocities rendering the variable mean flow index (Mx)	Perioperative
Ono et al. 21 (2012)	Cerebral blood flow (CBF) by TCD velocity, and near-infrared spectroscopy (NIRS) data	All enrolled patients underwent coronary artery bypass graft (CABG) surgery, valvular surgery, or both, which required CPB. NIRS regional cerebral oxygen saturation (rSCO2) monitoring with an INVOS monitor and bilateral TCD monitoring of the middle cerebral arteries (MCAs). A continuous, moving Pearson correlation coefficient was calculated regarding MAP and CBF velocity to generate the Mx and cerebral oxygenation index (COx).	Perioperative
Ono et al. 22 (2014)	rScO2 mesured through a NIRS monitor	Autoregulation was monitored during CPB, patients were connected to NIRS monitor. A continuous, moving Pearson correlation coefficient regarding the MAP and NIRS signals was then calculated to generate the variable COx	Perioperative
Semenyutin et al. 23 (2017)	Blood flow velocity (BFV) by TCD; ultrasound by Multidop TCD with 2 MHz transducers	Classified the patients with severe internal carotid artery stenosis according to preoperative state of cerebral autoregulation (CAR) and to assess its dynamics after surgery. The spectral amplitude of blood pressure (BP) and BFV derived from spectral power, phase shift (PS), and coherence coefficient were calculated in specifically selected range of systemic Mayer waves (M-waves) – transfer function analysis (TFA). This frequency band commonly reflects periodic BP fluctuations and is most informative for the assessment of the myogenic component of cerebrovascular response comparing with high-frequency oscillations	Preoperative and postoperative
Chan and Aneman 24 (2019)	NIRS monitor	The FORESIGHT Elite NIRS monitor was used to continuously measure the oxygen saturation of the cerebral tissue. CAR was assessed by the Pearson correlation regarding NIRS-derived cerebral tissue oxygen saturation and mean arterial pressure to derive the tissue oxygenation index. The MAP corresponding to a tissue oxygenation index threshold of 0.30 was also calculated to represent the lower (LLA) and upper (ULA) limits of autoregulation, and to calculate the range of intact autoregulation (LLA-ULA). The parameters evaluated were: optimal MAP, optimal tissue oxygenation index, LLA, ULA and range (autoregulation index, ARI)	Postoperative

It is important to highlight in Table 2 that CAR monitoring took place in different perioperative phases, which can limit the quality of the results found.

Of the 941 patients, 703 (74.70%) had preserved CAR, and 14 (1.5%) of these patients suffered a perioperative stroke. On the other hand, 238 (25.3%) experienced intraoperative impaired CAR, and 33 (3.5%) of these patients suffered a perioperative stroke.

**Figure 2:**
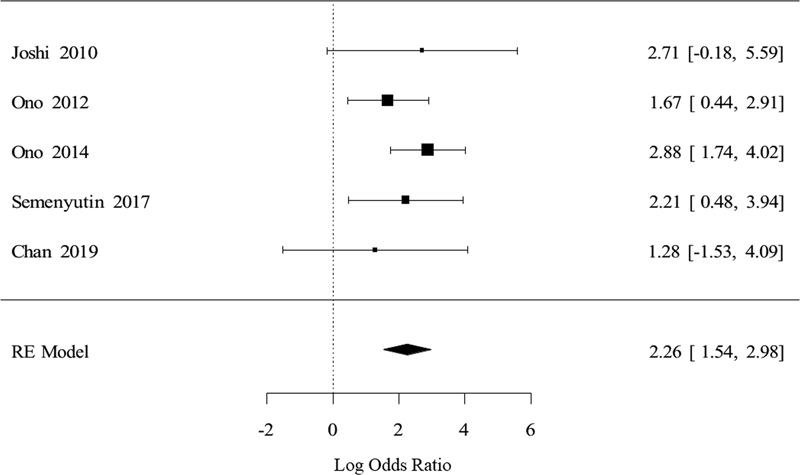
Forest plot displaying the individual OR log of the analyzed studies and the OR summary calculated through random-effects models. The squares in the forest plot are proportional to the sample size of each study.

**Figure 3:**
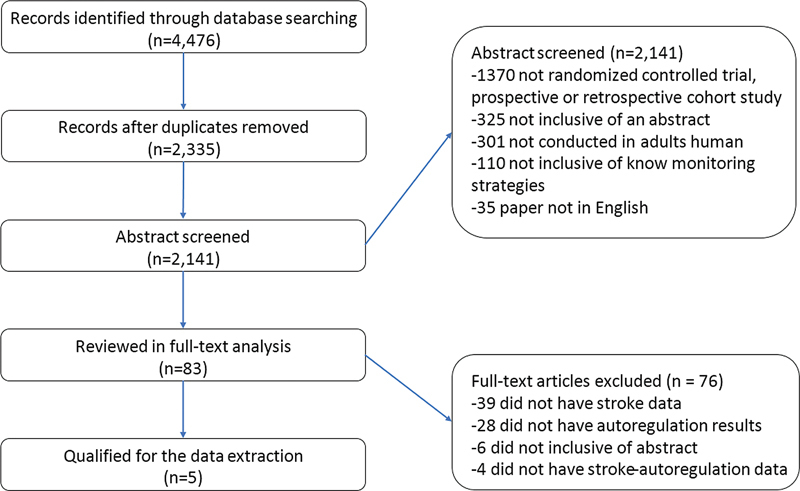
Funnel plot with an evaluation of the publication bias that presents a distribution with strong asymmetry.

The number of perioperative strokes was eight times higher among patients with intraoperative impaired CAR compare to those who had preserved intraoperative CAR. On the pooled analysis, impaired CAR was associated with stroke outcome (OR: 2.26; 95%CI: 1.54–2.98; *p* < 0.0001) ( Figure 2 ). There was no heterogeneity between the studies, which can be observed in the analysis I2 = 2.24% and also graphically visualized in Figure 3 .

In these studies, the procedures most commonly performed were cardiac surgeries (886: coronary artery bypass graft [CABG], CABG plus valve, and valve alone), carotid procedures (35; 3.7%) and other surgical procedures (20; 1.8%). These results showed that impaired CAR during intraoperative monitoring had a specificity of 77.07% (95%CI: 74.17–79.79%) and a sensitivity of 70.21% (95%CI: 55.11–82.66%) to predict perioperative stroke. However, these findings suggest that the odds of observing a stroke in a patient with impaired CAR under a major surgery is two times higher than in patients with preserved CAR.

## DISCUSSION

The primary aim of the present study was to determine whether monitoring impaired CAR can predict perioperative stroke. In general, after an evaluation of these results, we found that impaired CAR has a specificity of 77.07% and a sensitivity of 70.21% to predict perioperative strokes. In the present study, we observed impaired perioperative CAR in 25.3% of patients, and the odds of stroke occurring in a patient with impaired intraoperative CAR is 2 times higher than in patients with preserved intraoperative CAR. These results are in accordance with those of a previous study conducted by Caldas et al. 25 In the studies in this field, there are several methodological limitations regarding monitoring, the timing of the monitoring procedure, the interventions related and more parameters of neurological vulnerability. 26 Udesh et al. 17 showed that monitoring cerebrovascular hemodynamic parameters in association with electrophysiological modalities could increase the accuracy of the identification of perioperative stroke. We did not find studies investigating this association on multimodality monitoring. Parts of that data shed light on the risk factors for perioperative stroke. 27 Older age and preexisting cerebrovascular disease, especially white matter disease, are associated to a higher risk of perioperative neurological complications, including stroke. 25 Therefore, understanding the significance of impaired perioperative CAR may lead to the improvement of the models for the prediction of brain injury after surgery, and further investigation is required.

The impact of perioperative stroke on the outcome remains a great challenge in critical care. 28 Few institutions across the globe work on the immediate assessment of fast diagnosis and protocol-driven care. The previously mentioned “stroke code in the operating room” remains a challenge for the management of such a devastating disease. Therefore, understanding the mechanisms of cerebral hypoperfusion may play a major role in the correct assessment of the most sensitive tools for monitoring. Recently, Anetakis et al. 29 showed that this association may improve intraoperative stroke detection and time response for treatment with “last electric well time” definition.

The association of CAR impairment and stroke is better understood in acute brain injury. 25 Perioperative CAR impairment has already been associated with poor outcomes, 25 and the impact of cerebral hypoperfusion measured by TCD has already been demonstrated. 17 Nevertheless, the pathophysiology associated to this phenomenon has not been fully understood yet. The present systematic review and meta-analysis showed results that indicate that CAR impairment may play a role in the pathophysiology of perioperative stroke. There are reports 25 that perioperative stroke may occur in up to 5% to 10% of all surgical procedures. Moreover, this rate may be even higher in cardiovascular procedures. 25 These events could also be associated with higher mortality and incidence of neurological deficits. 28 30

The evidence suggests that hypoperfusion and embolic events may play a role in this setting, 17 28 with significant unfavorable outcomes and reduced quality of life, 28 31 32 with a three-fold increase in the rate of cognitive decline one year after the procedure. 28 However, few studies 28 31 32 address the potential causes of this devastating disease. The etiology of peroperative stroke is variable and not yet fully understood, and the present meta-analysis sheds some light into the comprehension of these mechanisms: CAR impairment, measured through different monitoring methods, was found to be associated with perioperative stroke in the studies reviewed.

But the mechanisms that trigger perioperative stroke remain largely unclear. However, cerebral hypoperfusion due to CAR impairment has been suggested as one of the main underlying causes. As aforementioned, different causes may be related, including microembolization, BP fluctuations a,nd CAR impairment. Other mechanisms have already been suggested in the literature. 33 34 Not all of those potential causes confirmed its implication. 33 35 Controlled studies 36 comparing the rate of adverse neurological outcomes in off-pump surgeries showed no statistical difference between another strategy.

Clinical information regarding the severity and location of the stroke would enable a very interesting analysis of perioperative CAR monitoring. The same relevance could be observed with a more robust list of clinical data from patients, including comorbidities, continuous drugs, and even clinical history. However, as expected, the present meta-analysis was limited to the data published in the studies analyzed, but we were able to show the prevalence of comorbidities on Table 1 .

Accordingly, although the limitations in terms of the extraction of these data and the pathogenesis of the neurological sequelae might be multifactorial, CAR impairment seems to be a key factor. However, no studies have described specific treatments that have been proven to overcome this important autonomic response. Nevertheless, studies 25 have shown that not only the identification of impaired CAR but also its burden (time spent outside the normal limits of pressure) are associated with stroke. Therefore, maintaining optimal cerebral perfusion during high-risk surgery seems to be important to prevent stroke, and it depends on the monitoring and management of CAR.

In conclusion, perioperative stroke remains a disease with a high burden and incidence of neurological complications. The mechanisms involved in it remain unclear, but CAR impairment seems to play a role. Many studies 25 have addressed the incidence and outcome impact of perioperative stroke. The present meta-analysis showed evidence of the impact of CAR impairment in the risk of developing perioperative stroke. There is already evidence showing that CAR impairment is involved in other perioperative complications. 5 Moreover, BP titration has been associated with better outcomes in patients with perioperative CAR monitoring. The present meta-analysis reinforces the role of CAR impairment in perioperative stroke and the need for more studies to elucidate CAR involvement and monitoring during surgeries. These data could, in the future, shed light on the best determination of CAR before, during and after surgery; disease-specific evaluation of the course of CAR over time and the quantification of the impact of CAR impairment on outcomes.
